# Diagnostic performance of intravoxel incoherent motion diffusion-weighted imaging and dynamic contrast-enhanced MRI for assessment of anal fistula activity

**DOI:** 10.1371/journal.pone.0191822

**Published:** 2018-01-25

**Authors:** Philippe Lefrançois, Mathieu Zummo-Soucy, Damien Olivié, Jean-Sébastien Billiard, Guillaume Gilbert, Juliette Garel, Emmanuel Visée, Perrine Manchec, An Tang

**Affiliations:** 1 Department of Radiology, Centre hospitalier de l’Université de Montréal (CHUM), Montréal, Québec, Canada; 2 MR Clinical Science, Philips Healthcare Canada, Markham, Ontario, Canada; 3 Centre Hospitalier Départemental de Vendée, Les Oudairies, La Roche-Sur-Yon, France; 4 Norimagerie, 1,3 chemin du Penthod, Caluire et Cuire, France; 5 Centre de recherche du Centre hospitalier de l’Université de Montréal (CRCHUM), Montréal, Québec, Canada; Henry Ford Health System, UNITED STATES

## Abstract

**Objective:**

To evaluate intravoxel incoherent motion (IVIM) diffusion-weighted imaging (DWI) and dynamic contrast-enhanced (DCE) magnetic resonance imaging (MRI) sequences for quantitative characterization of anal fistula activity.

**Methods:**

This retrospective study was approved by the institutional review board. One hundred and two patients underwent MRI for clinical suspicion of anal fistula. Forty-three patients with demonstrable anal fistulas met the inclusion criteria. Quantitative analysis included measurement of DCE and IVIM parameters. The reference standard was clinical activity based on medical records. Statistical analyses included Bayesian analysis with Markov Chain Monte Carlo, multivariable logistic regression, and receiver operating characteristic analyses.

**Results:**

Brevity of enhancement, defined as the time difference between the wash-in and wash-out, was longer in active than inactive fistulas (*p* = 0.02). Regression coefficients of multivariable logistic regression analysis revealed that brevity of enhancement increased and normalized perfusion area under curve decreased with presence of active fistulas (*p* = 0.03 and *p* = 0.04, respectively). By cross-validation, a logistic regression model that included quantitative perfusion parameters (DCE and IVIM) performed significantly better than IVIM only (*p* < 0.001). Area under the curves for distinguishing patients with active from those with inactive fistulas were 0.669 (95% confidence interval [CI]: 0.500, 0.838) for a model with IVIM only, 0.860 (95% CI: 0.742, 0.977) for a model with IVIM and brevity of enhancement, and 0.921 (95% CI: 0.846, 0.997) for a model with IVIM and all DCE parameters.

**Conclusion:**

The inclusion of brevity of enhancement measured by DCE-MRI improved assessment of anal fistula activity over IVIM-DWI only.

## Introduction

Anal fistulas are common, with an incidence of 8.6 per 100,000 population [1]. Underlying causes include Crohn's disease, pelvic infection, trauma, malignancy, and radiation therapy [2]. Anal fistulas may be assessed by physical examination under anesthesia, endoscopy, ultrasound, fistulography, computed tomography, and magnetic resonance imaging (MRI) [3–5]. Accurate characterization of fistulas is critical [6]. MRI has become a technique of choice for imaging anal fistulas because of its ability to identify tracts, define complex anatomy, and detect abscesses [4, 7–10]. Further, assessment of fistula activity plays a critical role in the selection of medical, surgical, or combined therapy [11–13] and patient outcome [4, 14, 15]. The detection of abscesses and fistula extensions by MRI can guide the surgeon to resect occult pathological structures that may be otherwise refractory to immunosuppressive therapy, potentially resulting in better patient outcomes [16].

MRI of anal fistulas is performed with multiple sequences [17]. T2-weighted sequences provide high contrast for anatomical assessment of the different layers of the anal sphincter, whereas gadolinium-enhanced T1-weighted sequences reveal areas with increased vascularity such as the wall of active fistulas and abscesses [4]. When interpreted in combination, T2-weighted [18] and gadolinium-enhanced T1-weighted sequences allow differentiation between fluid, inflammatory, and fibrotic tissues [4, 19–21]. Recent studies have suggested that diffusion-weighted imaging (DWI) sequences, which reflects motion of water molecules and their interactions with macromolecules and cell membranes, may be helpful for the diagnosis of anal fistulas [22, 23] and abscesses complicating anal fistulas by revealing restricted diffusion of water molecules due to viscous pus [24–26]. Considering the longer examination time, cost of contrast media injection, cost of supervision, and potential safety concerns, there is a need to determine whether DWI sequences are sufficient or whether contrast injection is required for assessment of anal fistula activity [27].

Assessment of MRI examinations is traditionally based on qualitative interpretation of signal characteristics. However, emergent techniques permit objective measurement of diffusion and perfusion parameters that may correlate with fistula activity. Intravoxel incoherent motion (IVIM)-DWI sequences assess perfusion fraction, extravascular molecular diffusion, and microcirculation [28]. Further, dynamic contrast-enhanced (DCE)-MRI allows semi-quantitative or quantitative assessment of tissue perfusion by continuously monitoring contrast media [29–32]. There is a need to identify quantitative parameters that predict anal fistula activity [33, 34], to assess the diagnostic accuracy of diffusion and perfusion sequences [35], and to examine the need for intravenous contrast injection in the assessment of anal fistula activity.

The purpose of this study was to evaluate IVIM-DWI and DCE-MRI sequences for quantitative characterization of anal fistula activity.

## Materials and methods

### Compliance with ethical standards

All procedures performed in studies involving human participants were in accordance with the ethical standards of the institutional and/or national research committee and with the 1964 Helsinki declaration and its later amendments or comparable ethical standards. This article does not contain any studies with animals performed by any of the authors. Informed consent was obtained from all individual participants included in the study. This retrospective, cross-sectional, single-site study was approved by our Institutional Review Board (Centre hospitalier de l’Université de Montréal (CHUM)).

### Study design and subjects

This retrospective, cross-sectional, single-site study was approved by our Institutional Review Board. Patients were included in this study if they had a clinical suspicion of anal fistulas between May 2011 and April 2015 at the Centre hospitalier de l’Université de Montréal (CHUM).

**Fig 1:**
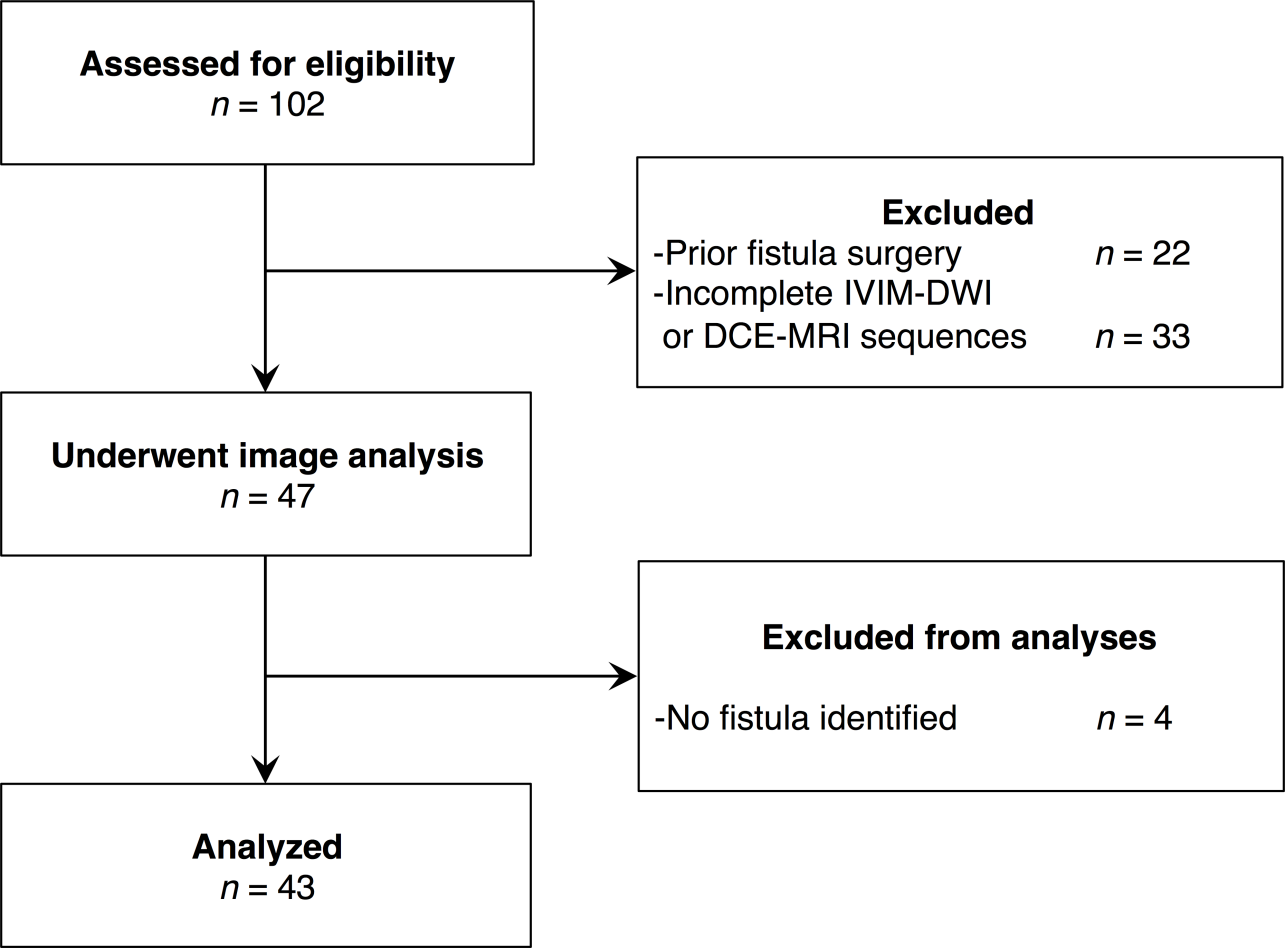
Study flowchart.

One hundred and two patients underwent MRI for clinical suspicion of perianal fistula (prior surgery, known inflammatory bowel disease, anal pain, anal abscess, pain with defecation, bloody or foul-smelling discharge in perianal area, etc.), using a consecutive recruitment scheme and signed an informed consent form. Twenty-two patients were excluded due to previous surgery for perianal fistula and 33 because their MRI studies did not include both the IVIM-DWI and DCE-MRI sequences. Forty-seven patients underwent image analysis and 4 were excluded because they had no visible fistula. Thus, this study included 43 patients with a visible fistula on MRI (**Fig 1**).

Patient demographics are summarized in **Table 1**and in **S1 Table**. Forty-nine percent (21 of 43) of patients were men. The median age of patients was 39 years. Etiologies of perianal disease were documented: 21 patients had known Crohn's disease, 1 had ulcerative colitis, 2 developed perianal fistula post-partum, 2 had suppurative hidradenitis, 1 had pilonidal sinuses, and 16 had idiopathic perianal fistula.

**Table 1:** Characteristics of subjects and observations included in study cohort.

Characteristic	Result
**Sex**	
Male	21/43 (49%)
Female	22/43 (51%)
**Age (y)**	
Median (range)	39 (20–55)
**Etiology of perianal disease**	
Crohn's disease	21/43 (49%)
Ulcerative colitis	1/43 (2%)
Post-partum disease	2/43 (5%)
Suppurative hydradenitis	2/43 (5%)
Pilonidal sinus	1/43 (2%)
Idiopathic perianal fistula	16/43 (37%)
**Time interval between MRI and reference standard (days)**	
Median	30
Interquartile range	20–60
**Reference standard**	
Surgery	9/43 (21%)
Clinical follow-up	34/43 (79%)

Numbers in parentheses are percentages, unless otherwise specified.

The median time interval between MRI and clinical reference standard was 30 days (interquartile range 20–60 days). The reference standard was surgical in 21% (9 of 43) cases and clinical follow-up in 79% (34 of 43) cases.

### MRI image acquisition

All acquisitions were performed on a 3T clinical system (Achieva TX, Philips Healthcare, Best, The Netherlands), using the integrated 2-channel body coil for signal transmission and a 16-channel body array for signal reception. Patients were imaged in supine position. The detailed MRI protocol and sequence parameters are summarized in **Table 2**.

**Table 2:** MRI protocol.

	T2w	T2w	T2w	T2w	IVIM-DWI	T1w Pre/Post	DCE-MRI
Sequence type	2D TSE	2D TSE	2D TSE	2D TSE	2D SE-EPI	2D TSE	3D GRE
Acquisition plane	Sagittal	Axial	Axial	Coronal	Axial	Axial	Axial
Fat suppression	-	-	SPAIR	SPAIR	SPIR	SPIR	SPAIR
Repetition time (msec)	3000	3435	3000	3000	3000	525	4.3
Echo time (msec)	80	80	70	70	53	10	2.2
Flip angle (degrees)	90	90	90	90	90	90	10
Bandwidth (Hz/pixel)	156.3	709.7	165.2	187.8	18.0	168.3	756.7
Echo train length	14	14	13	14	-	5	-
Slice thickness/Gap (mm)	4/0.4	4/1	4/1	4/1	6/0	4/1	3/0
Number of slices	23	38	38	32	26	38	81
Acquisition matrix	248 x 266	268 x 266	216 x 208	224 x 214	160 x 125	288 x 226	236 x 234
Reconstruction matrix	518 x 560	560 x 560	560 x 560	512 x 512	352 x 352	560 x 560	560 x 560
Field-of-View (mm)	200 x 216	216 x 216	216 x 216	200 x 200	340 x 340	216 x 216	216 x 216
Acceleration factor (SENSE)	-	-	2	2	1.6	2	1.5
Number of signal averages	3	2	3	3	4 b < 500 8 b > 500	3	1
b-values (sec/mm^2^)	-	-	-	-	0, 25, 50, 100, 150, 350, 550, 800	-	-
Acquisition time (min:sec)	5:48	4:28	5:00	4:48	5:39	4:21	0:22/dynamic for 21 dynamics

*DCE-MRI* = dynamic contrast-enhanced MRI. *GRE* = gradient-recalled echo. *IVIM-DWI* = intravoxel incoherent motion diffusion-weighted imaging. *SE-EPI* = spin echo echo planar imaging. SPAIR = spectral attenuated inversion recovery. *SPIR* = spectral presaturation with inversion recovery. *TSE* = turbo spin echo.

#### IVIM-DWI

IVIM was performed using a diffusion-weighted single-shot spin-echo echo planar imaging sequence with the following acquisition parameters: TR = 3000 ms, TE = 53 ms, flip angle = 90 degrees, FOV = 340 mm x 340 mm x 156 mm, spatial resolution at acquisition = 2.1 mm x 2.7 mm x 6 mm, spatial resolution at reconstruction = 1 mm x 1 mm x 6 mm, SENSE acceleration factor = 1.6, 8 b-values acquired for 3 orthogonal directions (b = 0, 25, 50, 100, 150, 350, 550, 800 sec/mm^2^), 4 signal averages for b < 500 sec/mm^2^ and 8 signal averages for b > 500 s/mm^2^. Mean acquisition time was 5 min 39 s.

#### DCE-MRI

Dynamic contrast-enhanced imaging (DCE-MRI) was performed using a dynamic 3D gradient-echo sequence with the following acquisition parameters: TR = 4.3 ms, TE = 2.2 ms, flip angle = 10 degrees, FOV = 215 mm x 215 mm x 165 mm, spatial resolution at acquisition = 0.85 mm x 0.85 mm x 6 mm, spatial resolution at reconstruction = 0.38 mm x 0.38 mm x 3 mm, SENSE acceleration factor = 1.5, 1 signal average. Imaging was performed for a total of 21 dynamics (1 prior and 20 after contrast injection) with a temporal resolution of 22.5 sec. Gadoteridol was administered intravenously at a dose of 0.1 mmol/kg (0.2 mL/kg) of body weight through an 18-gauge intravenous catheter with an automated injection pump (Optistar Elite, Mallinckrodt, Hazelwood, Mo). Injection of contrast medium was followed by a bolus of 15 mL saline solution at 2 mL/sec.

### Quantitative MRI analysis

IVIM-DWI.—IVIM-DWI analysis was performed using a segmented bi-exponential model [36]. The perfusion fraction *(f)* and diffusion coefficient *(D)* were first computed by performing a non-linear regression to a mono-exponential model using only images acquired with *b* ≥ 150 s/mm^2^. The perfusion coefficient (D*) was calculated in a subsequent step by performing a non-linear regression to a bi-exponential model using all *b* values and the previously calculated values for *f* and *D*. All IVIM analyses were performed in Matlab (R2012a, The Mathworks, Natick, USA). ROIs across multiple slices and using guidance from the different sequences were traced for each fistula, guided by raw MRI images on a separate workstation by an image analyst. Drawn ROIs encompassed the entire fistula, including its wall, lumen, and abscesses (if applicable) which are considered as indicators of activity [37]. To eliminate the potential confounding signal of tissue outside of the fistulas, the ROIs excluded fat in the ischiorectal fossa, perineal fascia, and subcutaneous fat as well as muscular components of the internal and external sphincters. Individual quantitative parameters, along with their standard deviation, were calculated by the algorithm for pixels included in the ROIs.

DCE-MRI.—Perfusion maps of semi-quantitative parameters (maximum relative enhancement, time of arrival, time to peak, wash-in rate, wash-out rate, brevity of enhancement (defined as the time difference between the wash-in and wash-out), area under the curve) were computed using a commercially available software package (Extended MR Workspace, Philips Healthcare, Best, The Netherlands). The area under curve was normalized by the background signal prior to contrast agent injection *(S*_*0*_*)*, the latter derived from a system of linear equations. Wash-in and wash-out rates were normalized using *S*_*0*_ in a scale-free manner. The original images and the calculated perfusion maps were then transferred to a dedicated analysis tool developed in Matlab for the region-of-interest (ROI) analysis. ROIs and their parameters were determined as described in the previous section, and included in **S1 Table**.

### Reference standard

Surgical assessment or clinical outcome were used as the reference standard to determine fistula activity. Surgical assessment referred to physical or intraoperative determination of fistula activity by a surgeon. Clinical outcomes referred to assessment of fistula activity based on symptoms such as pain, restriction of activities, and discharge [3]. Active fistulas were characterized by fluid drainage and signs of local inflammation, whereas inactive fistulas indicated absence of these features [3]. Since the clinical outcome was not standardized among surgeons and clinicians, we used a scale to determine disease activity: a score of 0 indicated the absence of fistula, 1 an inactive fistula, 2 an active fistula and 3 unknown activity, as per interpretation of clinicians’ and surgeons’ observations from patient chart review. The scale was further dichotomized into active (2) vs. inactive or unknown (1 or 3) fistula activity (based on clinical and/or surgical outcome), as our goal was to separate fistulas that were clearly active from those that were likely not.

### Blinding

The image analyst was blinded to the reference standard. The author (4 years of experience) who collected the clinical characteristics and reference standard was blinded to imaging results.

### Statistical analysis

Statistical analyses were performed by a bioinformatician (9 years of experience) using statistical software (R, version 3.2.5 including rjags, coda, pROC, OptimalCutpoints and MASS packages, R Foundation for Statistical Computing, Vienna, Austria). Categorical variables were expressed as numbers and percentages. Continuous variables were expressed as mean and standard deviation.

#### Quantitative analysis

Data from all IVIM-DWI and DCE-MRI parameters were obtained for each patient, taking into account a single fistula based on size (largest) when multiple fistulas existed in the same patient. Quantitative comparisons between inactive and active fistulas for IVIM-DWI and DCE-MRI parameters were performed using a Bayesian analysis with Markov Chain Monte Carlo (MCMC), with 100,000 iterations.

Multivariable logistic regression models were fitted from IVIM-DWI only and IVIM-DWI with DCE-MRI parameters, using the activity as the response variable, forced-entry for IVIM-DWI, and a bidirectional approach for DCE-MRI variables. Individual perfusion parameters that achieved or approached statistical significance were considered for simpler models using only IVIM-DWI with one or some selected parameters. ANOVA on nested models was performed to determine statistical significance of adding additional parameters based on the difference of deviance. *P* values were corrected for multiple hypothesis testing using the Holm-Bonferroni method.

#### Diagnostic accuracy

Receiver operating characteristics (ROC) curves were generated for logistic regression models based on quantitative IVIM-DWI only, IVIM-DWI and the most discriminant perfusion parameter, and IVIM-DWI with all DCE-MRI parameters, with the clinical reference standard as a reference. The area under the ROC curve was calculated for quantitative techniques. The diagnostic accuracy (sensitivity, specificity, accuracy, positive predictive value [PPV] and negative predictive value [NPV], along with 95% confidence intervals) of quantitative techniques was calculated for thresholds that maximized Youden's index. A bootstrapping, leave-one-out procedure was used for determining the confidence intervals (CI), with 2000 replicates.

## Results

### Quantitative analysis

**Fig 2:**
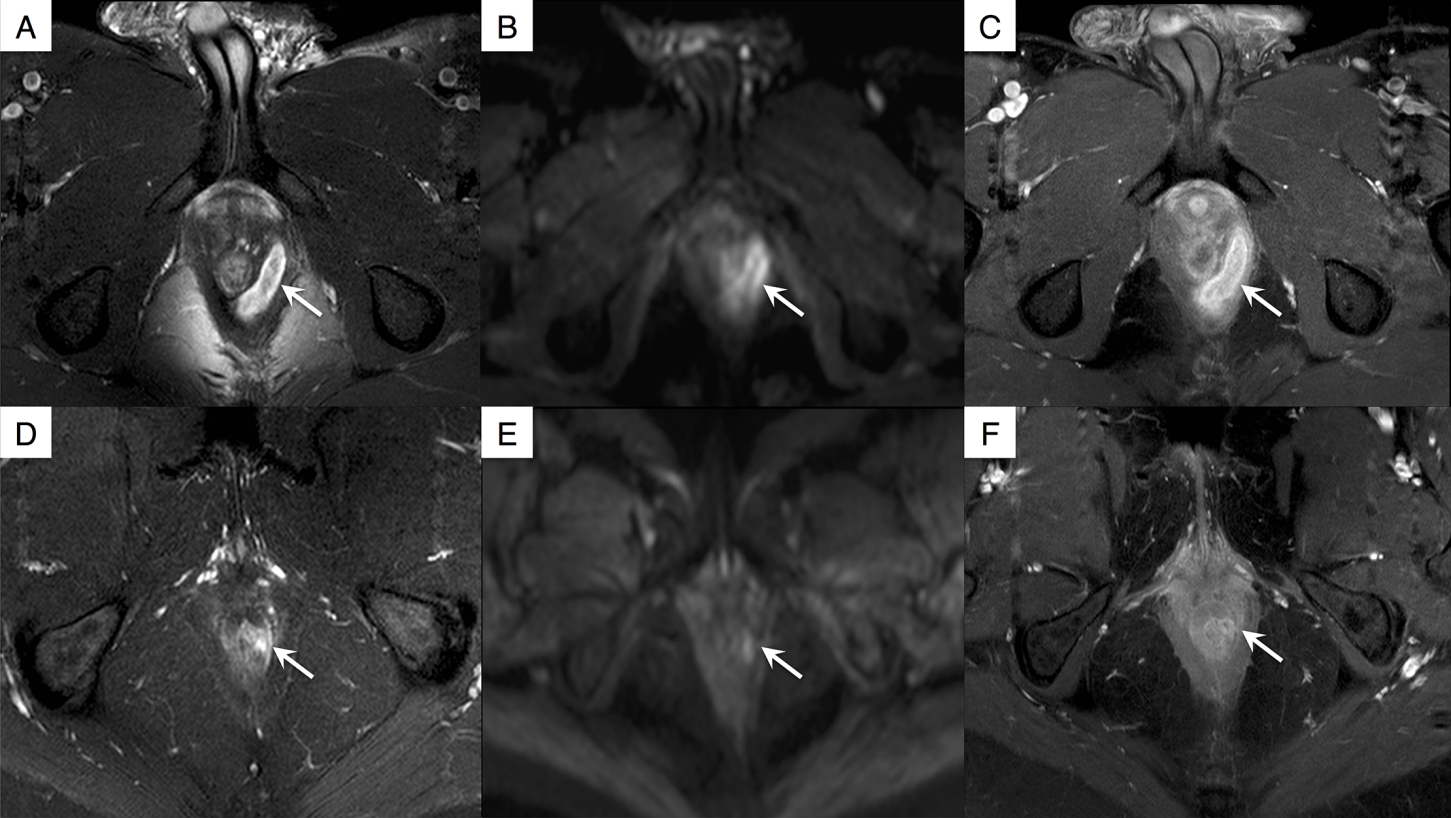
Representative images in 2 patients. Crohn's disease in a 21-year-old man with active fistula (arrows) shows (A) an inter-sphincteric abscess with marked hyperintense signal on the T2-weighted axial turbo spin echo sequence, (B) hyperintense signal on the IVIM-DWI sequence at *b* = 350 sec/mm^2^, and (C) marked enhancement on the contrast-enhanced sequence. Crohn's disease in a 46-year-old woman with inactive fistula (arrow) show (D) mild T2 hyperintense on the T2-weighted axial turbo spin echo sequence, (E) hypointense signal on the IVIM-DWI sequence at *b* = 350 sec/mm^2^, and (F) minimal enhancement on the contrast-enhanced sequence.

**Fig 3:**
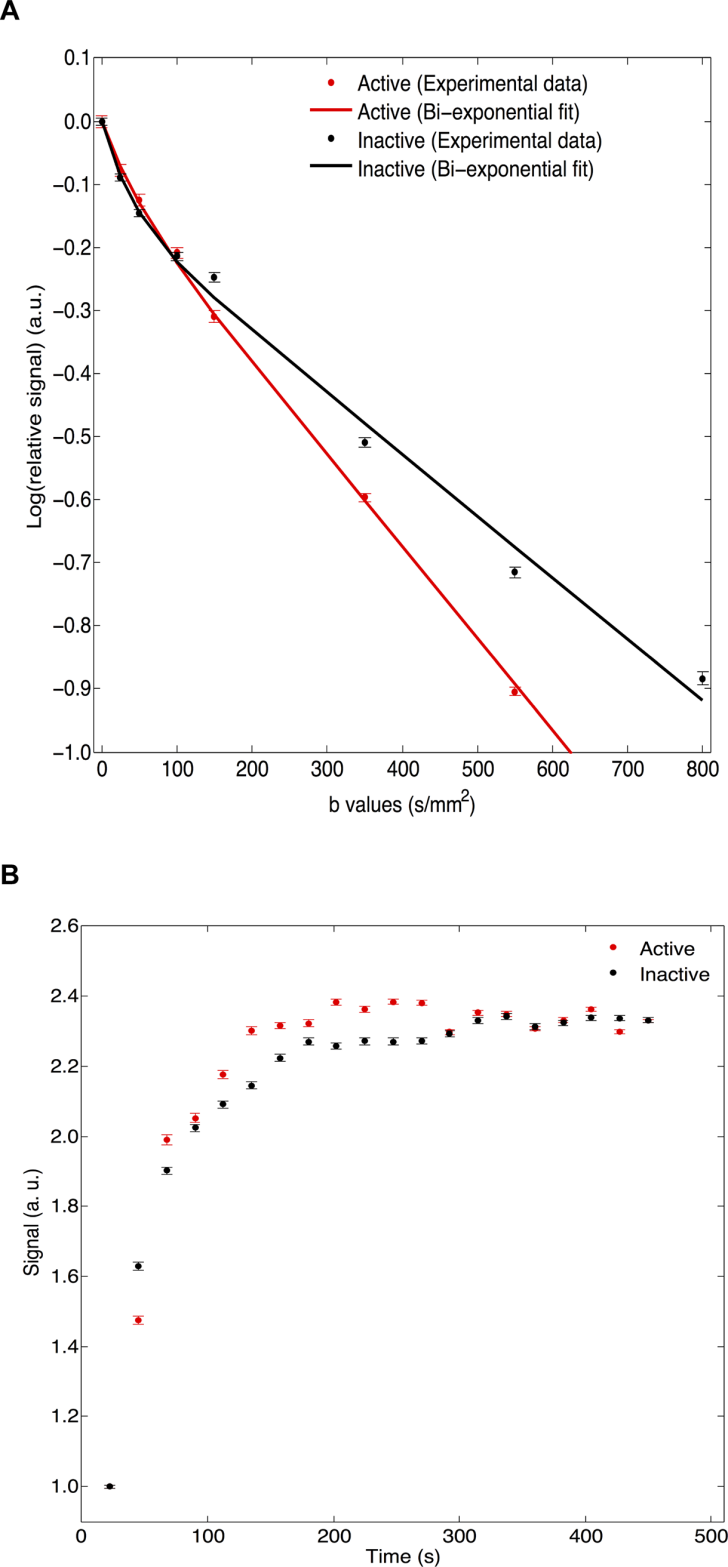
Quantitative IVIM-DWI and DCE-MRI analyses in 2 representative patients. Crohn's disease in a 21-year-old man with active fistula (results in red) and in a 46-year-old woman with inactive fistula (same patients as Fig 2). (A) IVIM-DWI signal versus *b*-value analysis with experimental data and segmented bi-exponential fit: in the active fistula, *D* was 1.24 x 10^−3^ sec/mm^2^, D* was 2.08 x 10^−2^ sec/mm^2^, and *f* was 0.07; in the inactive fistula, *D* was 1.18 x 10^−3^ sec/mm^2^, D* was 1.02 x 10^−2^ sec/mm^2^, and *f* was 0.14. (B) DCE-MRI analysis with experimental MRI signal intensity versus time data: in the active fistula, brevity of enhancement was 383.8 sec; in the inactive fistula, brevity of enhancement was 266.3 sec.

The IVIM-DWI and DCE-MRI parameters for 15 inactive and 28 active fistulas are summarized in **Table** 3. For the 3 IVIM-DWI parameters (D*, *D* and *f)*, none achieved statistical significance for discriminating activity of anal fistulas. Among the 7 DCE-MRI parameters, brevity of enhancement, defined as the time difference between the wash-in and wash-out, was significantly longer (331.9 ± 11.2 sec vs. 255.5 ± 15.2 sec; *P* = 0.02) and time-to-peak enhancement was longer (350.1 ± 12.8 sec vs. 314.7 ± 16.4 sec; *P* = 0.08) in active fistulas compared to inactive fistulas. The other DCE-MRI parameters were not significantly different between active and inactive fistulas. **Fig 2**illustrates representative images in active and inactive fistulas and **Fig 3**illustrates the corresponding quantitative analyses in active and inactive fistulas.

**Table 3:** Summary of quantitative and Semi-quantitative analyses.

Parameters	Inactive (*n* = 15)	Active (*n* = 28)	*P* value
IVIM-DWI			
*D* (x 10^−3^ mm^2^/sec)	1.20 ± 0.98	1.04 ± 0.52	0.79
D* (x 10^−2^ mm^2^/sec)	2.21 ± 1.07	1.90 ± 0.53	0.98
*f*	0.13 ± 0.02	0.12 ± 0.01	0.42
DCE-MRI			
Max relative enhancement (a.u.)	135.8 ± 12.3	120.2 ± 7.4	0.26
Time of arrival (sec)	18.5 ± 5.1	16. 7 ± 2.2	0.72
Time to peak (sec)	314.7 ± 16.4	350.1 ± 12.8	0.08
Wash-in rate (a.u.)	10.8 ± 0.9	13.0 ± 1.6	0.24
Wash-out rate (a.u.)	3.4 ± 0.5	3.3 ± 0.3	0.79
Brevity of enhancement (sec)	255.5 ± 15.2	331.9 ± 11.2	**0.02**
Normalized AUC (a.u.)	513.8 ± 60.8	493.6 ± 37.9	0.76

Numbers are mean ± standard deviation. *AUC* = perfusion area under curve. *DCE-MRI* = dynamic contrast-enhanced MRI. *IVIM-DWI* = Intravoxel incoherent motion imaging using diffusion-weighted imaging. *f* = perfusion fraction. *D* = diffusion coefficient. D* = perfusion coefficient.

The results of the multivariable logistic regression analyses are summarized in **Table** 4. The regression models with IVIM that included one perfusion parameter (brevity of enhancement) (adjusted *R*^*2*^ = 0.17; ANOVA on nested models *P* = 0.004) or IVIM with all DCE-MRI parameters (adjusted *R*^*2*^ = 0.39; ANOVA on nested models *P* < 0.001) provided a better fit to the activity of anal fistulas than IVIM only (adjusted *R*^*2*^ = 0.03).

**Table 4:** Multivariable logistic regression analysis.

Model	Parameter	Estimate	Standard Error	*P*	Adjusted *R*^*2*^
IVIM only	Intercept	4.82	2.06	0.019	0.03
	*D*	-2.80 X10^3^	1.58 X10^3^	0.076	
	D*	-13.6	31.1	0.662	
	*f*	-6.54	7.27	0.368	
IVIM and brevity of enhancement	Intercept	-0.215	2.80	0.939	0.17
	*D*	-4.01 X 10^3^	1.88 X 10^3^	0.033	
	D*	-40.5	34.9	0.246	
	*f*	-0.744	7.73	0.923	
	Brevity of enhancement	2.00 X 10^−2^	8.16 X 10^−3^	0.014	
IVIM and all perfusion parameters	Intercept	-37.9	23.1	0.101	0.39
	*D*	-1.45 X 10^4^	9.80 X10^3^	0.138	
	D*	33.8	64.5	0.601	
	*f*	-10.6	11.2	0.344	
	Brevity of enhancement	0.120	0.056	0.033	
	Normalized AUC	-4.54 X 10^−2^	2.24 X10^-2^	0.042	
	Max relative enhancement	0.166	0.090	0.066	
	Time of arrival	0.135	0.111	0.222	
	Time to peak	3.89 X10^-2^	3.63 X 10^−2^	0.284	
	Wash-in rate	0.480	0.308	0.119	
	Wash-out rate	-0.785	0.523	0.134	

*f* = perfusion fraction. *D* = diffusion coefficient. D* = perfusion coefficient.

The regression coefficient estimates for D*, *D*, and *f* were somewhat similar in the model with IVIM only (-13.6, -2.80 X 10^3^, and -6.54, respectively), the model with IVIM and brevity of enhancement (-40.5, -4.01 X 10^3^, and -0.744, respectively), and the model with IVIM and all perfusion parameters (33.8, -1.45 X 10^4^, and -10.6, respectively).

In the model with IVIM and brevity of enhancement, higher brevity of enhancement was significantly associated with fistula activity (*P* = 0.014). In the model with IVIM and all perfusion parameters, enhancement was also the main parameter associated with fistula activity (*P* = 0.033).

### Diagnostic accuracy

**Fig 4:**
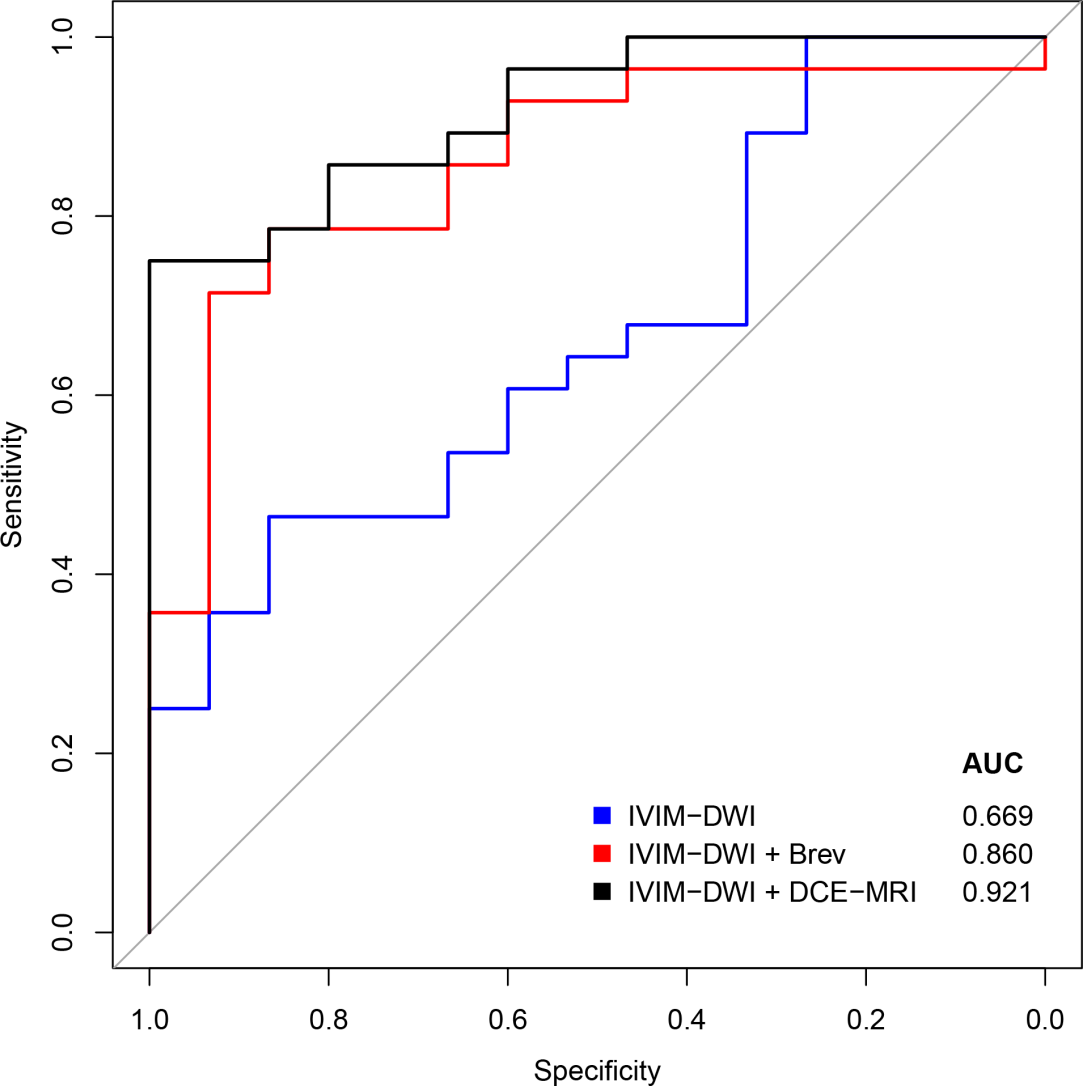
ROC curves. **ROC** curve analysis of quantitative analysis of IVIM-DWI, IVIM-DWI and brevity of enhancement, and IVIM-DWI and DCE-MRI (all parameters).

Receiver operating characteristics (ROC) curve analysis is provided in **Fig 4**and area under the ROC curve, sensitivity, specificity, PPV, and NPV are provided in **Table** 5.

**Table 5:** Diagnostic performance of quantitative models.

Analyses	AUC	Sensitivity (%)	Specificity (%)	Accuracy (%)	PPV (%)	NPV (%)
IVIM-DWI	0.669 [0.500, 0.838]	46 (13/28) [28, 66]	87 (13/15) [60, 98]	60 (26/43) [51, 81]	87 (13/15) [60, 94]	46 (13/28) [28, 89]
IVIM-DWI and brevity of enhancement	0.860 [0.742, 0.977]	79 (22/28) [59, 92]	87 (13/15) [60, 98]	81 (35/43) [72, 93]	92 (22/24) [71, 97]	68 (13/19) [46, 95]
IVIM-DWI and DCE-MRI	0.921 [0.846, 0.997]	75 (21/28) [55, 89]	100 (15/15) [78, 100]	84 (36/43) [77, 95]	100 (21/21) [83, 100]	68 (15/22) [47, 100]

*AUC* = area under curve for ROC. *IVIM-DWI* = Intravoxel incoherent motion imaging using diffusion-weighted imaging. *DCE-MRI* = dynamic contrast-enhanced MRI. *PPV* = positive predictive value. *NPV* = negative predictive value.

To distinguish active from inactive fistulas, the model with IVIM only had an AUC of 0.669 (95% CI: 0.500, 0.838) and provided 46% sensitivity, 87% specificity, 87% PPV, and 46% NPV; the model with IVIM and brevity of enhancement had an AUC of 0.860 (95% CI: 0.742, 0.977) and provided 79% sensitivity, 87% specificity, 92% PPV, and 68% NPV; and the model with IVIM and all DCE-MRI parameters had an AUC of 0.921 (95% CI: 0.846, 0.997) and provided 75% sensitivity, 100% specificity, 100% PPV, and 68% NPV.

To distinguish active from inactive fistulas, the quantitative model with IVIM only had an accuracy of 60% whereas models that included brevity of enhancement or all perfusion parameters had accuracies of 81% and 84%, respectively.

## Discussion

This retrospective clinical study evaluated the diagnostic performance of IVIM-DWI and DCE-MRI sequences for quantitative assessment of anal fistula activity, using clinical outcome or surgical assessment as the reference standard. All patients included had an anal fistula visible on MRI. Double-blinding was applied between quantitative MRI analyses and documentation of the clinical reference standard.

We found that active fistulas had a longer brevity of enhancement compared to inactive fistulas, which reflects a longer time between wash-in and wash-out of contrast agent on the time-intensity curves [38]. This is consistent with the empirical observation that active fistulas show persistent enhancement after contrast injection [33, 39]. We hypothesize that wash-in was faster in active fistulas presumably due to increased permeability from inflammation, while wash-out was slow in both inactive (due to fibrous scar) and active (due to inflammatory exudate) fistulas. On an individual basis, no other quantitative IVIM-DWI or DCE-MRI parameters significantly discriminated between active and inactive fistulas. We also found higher diagnostic accuracy of logistic regression models that included one or several DCE-MRI parameters in addition to IVIM for detection of active fistulas. These findings confirm that intravenous contrast injection is helpful for assessment of anal fistula activity. In complex cases with multiple tracts, the inclusion of contrast-enhanced perfusion sequences may help identify active tracts; select medical or surgical treatment; and guide the choice of setons, fibrin glue, collagen plugs, or flaps to cover the internal opening of active fistulas [12].

To distinguish active from inactive fistulas, we found that models that included at least one perfusion parameter provided 87% or greater specificity and 81% or greater accuracy. Considering the small incremental improvement in specificity between the model with a single perfusion parameter and a model that included all perfusion parameters, an approach based on assessment of brevity of enhancement may suffice to distinguish active fistulas.

Dynamic contrast-enhanced techniques have been studied for assessment of anal fistula activity [33, 40, 41]. Early studies relied on qualitative assessment [40], whereas more recent studies have assessed the correlation between fistula activity with semi-quantitative (analysis of the parameters of the time-intensity curves) [33] or quantitative (complex pharmacokinetic modeling) analysis [41]. Horsthuis *et al* [33] found a weak correlation between a perianal disease activity index and an MRI-based score of disease activity developed by Van Assche *et al*. [37] that takes into account the number of fistula tracks, location, extension, T2 hyperintensity, presence of collections, and rectal wall involvement. However, no significant differences were observed between MRI scores before and after therapy. Ziech *et al* showed that perianal disease activity index correlated moderately with maximum enhancement, initial slope of increase, and volume of enhancing pixels [41]. Further, they found that volume transfer constant *(K*^*trans*^*)* values decreased with anti-TNF-α treatment [41]. Unlike this latter study, we have not designed ours to include pharmacokinetic modeling, which is not performed in routine clinical care.

Diffusion-weighted imaging sequences have recently been studied for the assessment of anal fistulas [22–24]. Hori *et al* [22] found that DWI improved the visualization of internal opening, external opening, extent of fistulas, and overall confidence to the interpretation. However, they found no significant difference in detection sensitivity when comparing contrast-enhanced and T2-weighted images versus DWI-MRI and T2-weighted images. Dohan *et al* found increased conspicuity of fistulas in DWI and significantly lower ADC values in abscesses, but no correlation between fistula activity and ADC values [24]. Similarly, Yoshikazo et *al* found lower ADC in active than inactive fistulas, unlike our results which revealed no IVIM-DWI parameters that could significantly discriminate activity of anal fistulas [23]. However, all their patients have received antibiotics before the MR examination, which may have influenced fistula activity and the interpretation of ADCs. Of note, all prior studies that evaluated DWI were based on subjective interpretation of diffusion-weighted images [22] or on mono-exponential analysis of ADC [23, 24].To our knowledge, this is the first to assess quantitative parameters based on IVIM-DWI analysis. This is pertinent because IVIM-DWI addresses biomarkers of perfusion fraction, extravascular molecular diffusion, and microcirculation that are related to physiological phenomena [23, 28].

### Limitations

Our study has some limitations. First, in this retrospective study, the median time interval between MRI and the clinical reference standard was 30 days. Same-day comparison between an index test and reference standard is preferable, but logistically more challenging to achieve. However, we anticipate that this lag provides a conservative estimate and that contemporaneous imaging would have improved the diagnostic accuracy. MRI scanning time was also increased compared to standard MRI evaluation of perianal fistulas.

Second, physicians (gastroenterologists or surgeons) were not blinded to the results of the qualitative MR interpretation, as they had access to the imaging report. However, they were blinded to quantitative analysis presented in this study. Thus, double-blinding was preserved for quantitative results. The gold standard depended on physicians’ clinical and/or surgical evaluation, which may have incorporated qualitative MR imaging reports in decision making. In addition, MR imaging has the potential to better characterize complex fistula tracts than clinical assessment alone. In these select cases, it is possible that clinical evaluation may constitute a suboptimal reference standard.

Third, drawn ROIs encompassed the entire fistula, including the wall, lumen, and abscesses. Hence, ROIs may have included both active and inactive portions of the same fistula. Further, because fistulas have variable lengths and their walls variable thickness, the semi-quantitative parameters derived in this study represent averages of the entire fistula. Although global ROI placement may be viewed as a technical limitation, this approach eliminated subjectivity associated with ROI placement in portions of the fistula.

## Conclusions

In conclusion, this retrospective cross-sectional study showed that contrast-enhanced MRI may help determine activity of anal fistulas. In our study, analysis of DCE-MRI signal-intensity curves revealed that brevity of enhancement provided separation between inactive and active anal fistulas. The inclusion of brevity of enhancement measured by semi-quantitative DCE-MRI achieved greater diagnostic accuracy for classification of anal fistula activity than IVIM-DWI only. These results suggest that future studies should take into account analysis of perfusion to provide an objective assessment of anal fistula disease activity. Future work may explore quantitative analysis of DCE-MRI to produce parameters related to pathophysiological properties of anal fistulas.

## Supporting information

S1 Table Clinical features and analytical parameters from 43 patients with fistulas included in this study. (XLSX)

## Abbreviations

ANOVA: analysis of variance
AUC: area under curve
CI: confidence interval
DCE: dynamic contrast-enhanced
DWI: diffusion-weighted imaging
IVIM: intravoxel incoherent motion
MRI: magnetic resonance imaging
NPV: negative predictive value
PPV: positive predictive value
ROC: receiver operating characteristic
